# Association of sleep quality with lower urinary tract symptoms/benign prostatic hyperplasia among men in China: A cross-sectional study

**DOI:** 10.3389/fnagi.2022.938407

**Published:** 2022-10-24

**Authors:** Yifan Li, Xianghong Zhou, Shi Qiu, Boyu Cai, Sheng Wang, Lei Chen, Dan Hu, Zhongyuan Jiang, Mingda Wang, Xingyu Xiong, Kun Jin, Qiang Wei, Lu Yang, Li Ma

**Affiliations:** ^1^Department of Urology, Institute of Urology, National Clinical Research Center for Geriatrics and Center of Biomedical Big Data, West China Hospital of Sichuan University, Chengdu, China; ^2^West China School of Clinical Medicine, West China Hospital, Sichuan University, Chengdu, China; ^3^Department of Clinical Research, West China Hospital, Sichuan University, Chengdu, China; ^4^Institute of Hospital Management of West China Hospital/West China School of Nursing, Sichuan University, Chengdu, China; ^5^The First People’s Hospital of Longquanyi District, Chengdu, China

**Keywords:** sleep disorder, WCNPCS, LUTS/BPH, risk factor, Pittsburgh sleep quality index (PSQI)

## Abstract

**Objective:**

As the population aged, voiding dysfunction has been steadily rising among males during the past decade. Increasing evidence showed that sleep disorders are associated with an increasing risk of various diseases, but the association between sleep disorders and lower urinary tract symptoms (LUTS)/benign prostatic hyperplasia (BPH) among Chinese males have not been well characterized.

**Materials and methods:**

We conducted a cross-sectional analysis of data from West China Natural Population Cohort Study (WCNPCS) 2019–2021. Sleep quality was assessed by Pittsburgh sleep quality index (PSQI) in Chinese version. LUTS/BPH as a dependent variable of a binary variable, assessed by a self-reported questionnaire. Multivariate logistic regression analysis were performed to evaluate the correlation between sleep disorders and the risk of LUTS/BPH after adjusting for confounding factors.

**Results:**

11,824 eligible Chinese men participated in this cross-sectional survey. In multivariate logistic regression analysis, after adjusting for confounding variables, global PSQI score (OR: 1.257, 1.119–1.411, *p* < 0.001) and its six compounds (Subjective sleep quality: OR: 1.376, 1.004–1.886, *p* = 0.048; Sleep latency: OR: 0.656, 0.557–0.773, *p* < 0.001; Sleep duration: OR: 1.441, 1.189–1.745, *p* < 0.001; Habitual sleep efficiency: OR: 1.369, 1.193–1.570, *p* < 0.001; Daytime dysfunction: OR: 1.702, 1.278–2.267, *p* < 0.001) except the use of sleep drug subgroup were significantly positively correlated with LUTS/BPH prevalence. Significant interaction effects were observed in age subgroups (age-young group: age < 51; age-middle group: 51 ≤ age ≤ 61; age-older group: age > 61) (*P* < 0.05). Among older participants, sleep disorders were more significantly associated with the risk of LUTS/BPH.

**Conclusion:**

There was a significant association between poor sleep quality and increased prevalence of LUTS/BPH, especially among the elderly male population, suggesting an important role of healthy sleep in reducing prostate disease burden.

## Introduction

Benign prostatic hyperplasia (BPH) is the most common benign disease among the causes of micturition disorders in elderly men. It usually occurs after the age of 40. The prevalence rate of men over the age of 50 is 50%, and it is as high as 83% at the age of 80 (Berry et al., 1984; Gu et al., 1994). Wang et al. (2015) showed that the prevalence of BPH in Chinese population gradually increased from 2.9 to 69.2% among 40–80 years old. It is obvious that the prevalence of BPH has increased sharply with the age of Chinese men. The high prevalence of BPH greatly increases the health and economic burden of our society and the pain of these patients (Speakman et al., 2015).

Benign prostatic hyperplasia is due to the proliferation of stromal cells and epithelial cells stimulated by hormones and their active metabolites, causing the formation of anatomical hypertrophy prostate, which is manifested as urodynamic bladder outlet obstruction (BOO), resulting in lower urinary tract symptoms (LUTS), such as urinary retention, weak urine flow, etc. (Chughtai et al., 2016; Pejčić et al., 2017). In this study, LUTS/BPH refers to LUTS mainly manifested as obstruction caused by BPH.

Some studies have confirmed that sleep ensures the completion of important physiological functions by promoting the development of the central nervous system and the recovery of physical functions, which is a key factor in maintaining health (Tufik et al., 2009; Li et al., 2021). Researches involving both animals and humans have shown that sleep restriction can cause cognitive, immune, metabolic and hormonal disorders (Spiegel et al., 1999; Everson and Crowley, 2004; Hipólide et al., 2006). Previous cohort studies conducted by Koskderelioglu et al. (2017) and Branche et al. (2018) showed that poor sleep quality was associated with prostate disease.

However, the correlation between sleep quality and the risk of LUTS/BPH in Chinese men remains uncertain. Therefore, the purpose of this study is to analyze the potential association between sleep quality (measured by PSQI score) and LUTS/BPH in Chinese men. Importantly, the results may support the discovery of new strategies to prevent LUTS/BPH and improve the quality of life of aging population.

## Materials and methods

### Study population

The current research is a cross-sectional analysis based on the data of the West China Natural Population Cohort Study (WCNPCS) collected from May 2019 to June 2021. The sampling method of this study is cluster sequential sampling and the data was collected from three regions of Sichuan Province, the most populous province in Western China, including Mianzhu, Longquan, and Pidu. This study aimed to establish a large-scale prospective follow-up natural population cohort and collect various information of community participants in order to evaluate the health status of the general population in Western China.

**FIGURE 1:**
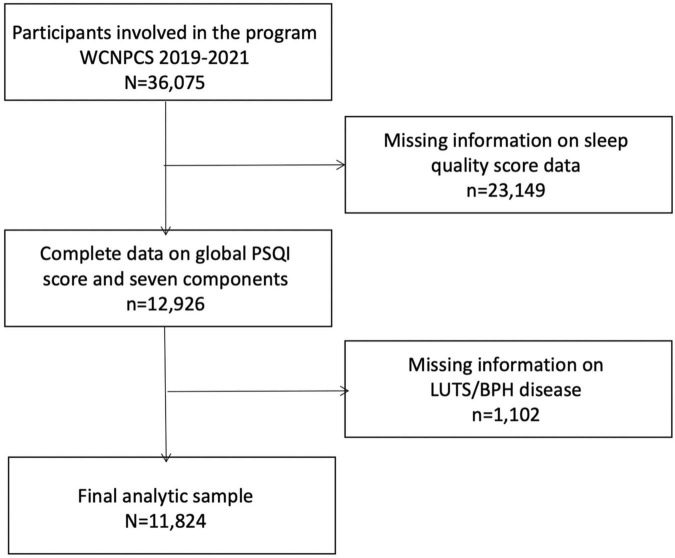
Flowchart of participant inclusion and exclusion for analysis.

A total of 36,075 participants aged 18–65 were included in this study. Participants were further excluded for lack of sleep quality and LUTS/BPH disease information (*n* = 1,102) and sleep quality score data (*n* = 23,149). Our final analysis sample was 11,824 participants (Figure 1). Specific general information (e.g., demographics, social-economic, education level and physical activities) was obtained through face-to-face interviews. Participants were further recruited for physical examination to collect biological samples, which were conducted by trained medical personnel in specially equipped mobile examination centers (MECs). Participants were recruited on a voluntary basis, and each participant signed and obtained informed consent before the survey. All research protocols were in accordance with the 1975 Helsinki Declaration and the applicable amendments at the time of the survey. The study protocol was approved by the ethics committee of West China Hospital of Sichuan University. The study was registered in China Clinical Trial Registration Center (Registration No. ChiCTR1900024623, 2019/07/19).

### Measurement of sleep quality

A PSQI questionnaire translated into Chinese was used to evaluate sleep quality. It is a standard self-report, including a 19-item questionnaire designed to collect a person’s subjective feelings about sleep habits for more than 1 month (Buysse et al., 1989). Each item is divided into four levels, with scores ranging from 0 to 3. PSQI has been used to diagnose sleep disorders in many clinical applications and has been proved to have good reliability, validity, and sensibility (Tsai et al., 2005; Mollayeva et al., 2016). It estimates several different aspects of sleep, which affect seven aspects of sleep problems, including subjective sleep quality, sleep latency, sleep duration, habitual sleep frequency, sleep disorders, use of sleep drugs, and daytime dysfunction (Buysse et al., 1989). The sum constitutes the global sleep quality score (ranging from 0 to 21), and the higher score mean the worse sleep quality. The global PSQI score is divided by 7 points, which can distinguish poor or good sleep. It has high diagnostic sensitivity and specificity in Chinese population (98.3 and 90.2% respectively) (Li et al., 2021).

### Measurement of lower urinary tract symptoms/benign prostatic hyperplasia

In addition, males were asked, “Have you ever been diagnosed with prostate hyperplasia?” Related symptoms of prostatic hyperplasia, including difficulty urinating, increased nocturia, urinary incontinence, were explained to all the participants. In WCNPCS study, symptoms were mainly assessed based on participant self-report, which was also commonly used in previous studies (Verhamme et al., 2002; Xiong et al., 2020; Zhang et al., 2022). The current study showed that men with sleep disorders are more likely to report daytime LUTS (Fantus et al., 2018). In order to make the results more reliable and exclude the direct impact of nocturia on sleep, We introduced daytime LUTS, which means symptoms of LUTS/BPH except nocturia.

### Covariates

Information about sociodemographic characteristics and lifestyle factors was collected through questionnaire survey. For continuous covariates including age (year), body mass index (BMI, kg/m2), waist-to-hip ratio (WHR), the patient health questionnaire-9 (PHQ-9), generalized anxiety disorder-7 (GAD-7) and creatinine (Cr μmol/L). For categorical covariates including education level (primary school, junior school, high school college, or graduate), marital status (married, unmarried, divorced, or widowed), smoking status (current, occasionally, ever or never), drinking status (yes, ever or no), coffee and tea intake (1–2 times/week, 3–5 times/week or >5 times/week), comorbidity index, diabetes mellitus (DM) (yes, prediabetes or no) and physical activity (sufficient, not sufficient or inactive). Diabetes mellitus, congestive heart failure, coronary artery disease, chronic obstructive pulmonary disease (chronic bronchitis and/or emphysema) and hypertension, cancer consisted of comorbid conditions. The number of subjects reported conditions were then combined to generate an ordinal comorbidity index. Exclusive diabetes was excluded and the total number of reported diseases was merged to create a sequential comorbidity index (Fantus et al., 2018). Individuals with a PHQ-9 score ≥ 10 are considered to have depressive symptoms (Kroenke et al., 2001). Calculate the average intake of coffee or tea in the dietary interview in a week to indicate the intake of caffeine (times/week, classification).

### Statistical analysis

In this study, continuous variables were presented as mean ± standard deviation, while categorical variables were presented as proportions. Chi-square (or Fisher’s exact test) analysis was used to assess categorical variables and *t*-test analysis was used to assess continuous variables. Multivariate logistic regression analysis was used to examine the odds ratio (or) and 95% confidence interval (CI) of the risk of LUTS/BPH. The independent variable in this study is the presence or absence of LUTS/BPH status, and sleep quality (PSQI global score ≤ 7 or >7) and seven components of PSQI were used as independent variables. ORs and 95% CI were calculated. Meanwhile, we regard the PSQI global score as a continuous variable and conduct multiple logistic regression again as a sensitivity analysis. The ORs were adjusted for age in the minimum adjustment model (Model I). In the fully-adjusted model (Model II), the ORs is adjusted for age, BMI, educational level, marital status, smoking status, drinking status, coffee intake, tea intake, WHR, PHQ-9, GAD-7, comorbidity index, DM, physical activity and Cr. We repeated the above analysis in daytime LUTS to reduce the impact of nocturia on the results. In order to explore whether this association was modified by other confounding factors, we conducted subgroup analyses. The statistical software packages R^1^ (The R Foundation) and EmpowerStats^2^ (X&Y Solutions, Inc., Boston, MA, United States) were used in the above statistical analyses. A *p*-value < 0.05 was considered statistically significant.

## Results

### Study population

A total of 11,824 participants were included in the study. Table 1 details the baseline characteristics of participants. The LUTS/BPH group was older, with higher WHR, lower education level, higher anxiety index, more obvious depression, easier smoking, more tea intake, higher comorbidity index, and inactive outdoor sports. In addition, there were significant differences in creatinine concentration, marital status, drinking, and coffee intake in the group with LUTS/BPH (*P* < 0.005).

**TABLE 1:** Baseline characteristics of participants.

Variable	Total	LUTS/BPH		*P*-value
	(*N* = 11,824)	Yes (*n* = 2,901)	No (*n* = 8,923)	
Age (year)	57.81 ± 12.40	60.51 ± 11.81	56.93 ± 12.46	<0.001
BMI (kg/m^2^)	24.79 ± 3.21	24.76 ± 3.17	24.80 ± 3.22	0.676
WHR	0.89 ± 0.06	0.89 ± 0.06	0.88 ± 0.06	<0.001
PHQ-9	0.814 ± 2.00	0.94 ± 2.19	0.78 ± 1.93	<0.001
GAD-7	0.76 ± 2.09	0.85 ± 2.27	0.73 ± 2.03	<0.001
Cr (μmoI/L)	72.62 ± 24.19	71.91 ± 15.80	72.85 ± 26.35	0.002
Educational level				<0.001
Primary school	3,371 (34.16%)	985 (41.65%)	2,386 (31.80%)	
Junior school	3,785 (38.36%)	912 (38.56%)	2,873 (38.29%)	
High school	1,543 (15.64%)	307 (12.98%)	1,236 (16.47%)	
College	1,144 (11.59%)	157 (6.64%)	987 (13.16%)	
Graduate	25 (0.25%)	4 (0.17%)	21 (0.28%)	
Marital status				<0.001
Married	11,006 (93.83%)	2,680 (93.32%)	8,326 (93.99%)	
Unmarried	190 (1.62%)	29 (1.01%)	161 (1.82%)	
Divorced	209 (1.78%)	54 (1.88%)	155 (1.75%)	
Widowed	136 (1.16%)	47 (1.64%)	89 (1.01%)	
Smoking status				<0.001
Current	5,053 (42.79%)	1,267 (43.68%)	3,786 (42.51%)	
Occasionally	352 (2.98%)	69 (2.38%)	283 (3.18%)	
Ever	1,541 (13.05%)	450 (15.51%)	1,091 (12.25%)	
Never	4,862 (41.18%)	1,115 (38.44%)	3,747 (42.07%)	
Drinking status				0.044
Yes	6,072 (51.43%)	1,445 (49.83%)	4,627 (51.95%)	
Ever	868 (7.35%)	238 (8.21%)	630 (7.07%)	
No	4,866 (41.22)	1,217 (41.97%)	3,649 (40.97%)	
Coffee drinking				<0.001
1–2 times per week	752 (6.37%)	141 (4.86%)	611 (6.86%)	
3–5 times per week	75 (0.64%)	10 (0.35%)	65 (0.73%)	
>5 times per week	74 (0.63%)	21 (0.72%)	53 (0.60%)	
No	10,901 (92.37%)	2,727 (94.07%)	8,174 (91.81%)	
Tea drinking				<0.001
1–2 times per week	1,699 (14.40%)	350 (12.07%)	1,349 (15.15%)	
3–5 times per week	925 (7.84%)	186 (6.42%)	739 (8.30%)	
>5 times per week	5,147 (43.61%)	1,320 (45.53%)	3,827 (42.99%)	
No	4,031 (34.16%)	1,043 (35.98%)	2,988 (33.56%)	
Comorbidity index				<0.001
0	5,737 (48.53%)	1,256 (43.30%)	4,481 (50.24%)	
1	5,646 (47.76%)	1,500 (51.71%)	4,146 (46.48%)	
2	422 (3.57%)	139 (4.79%)	283 (3.17%)	
3	16 (0.14%)	6 (0.21%)	10 (0.11%)	
DM				0.005
Yes	893 (7.58%)	259 (8.97%)	634 (7.13%)	
Prediabetes	147 (1.25%)	36 (1.25%)	111 (1.25%)	
No	10,739 (91.17%)	2,594 (89.79%)	8,145 (91.62%)	
Physical activity				<0.001
Sufficient	5.877 (49.77%)	1,470 (50.67%)	4,407 (49.48%)	
Not sufficient	1,550 (13.13%)	303 (10.45%)	1,247 (14.00%)	
Inactive	4,381 (37.10%)	1,128 (38.88%)	3,253 (36.52%)	

BMI, body mass index; WHR, waist-to-hip ratio; PHQ-9, the patient health questionnaire-9; GAD-7, generalized anxiety disorder-7; Cr, creatinine; DM, diabetes mellitus.

### Association between sleep quality and lower urinary tract symptoms/benign prostatic hyperplasia

**FIGURE 2:**
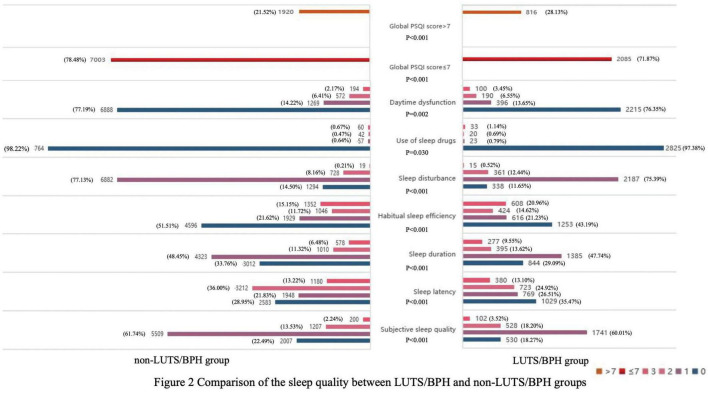
Comparison of the sleep quality between LUTS/BPH and non-LUTS/BPH groups.

We compared the distribution of sleep quality between the two groups with and without LUTS/BPH (Figure 2). In the LUTS/BPH group with a global PSQI score higher than 7, and this group showed higher score, which means worse sleep quality in six aspects: objective sleep quality, sleep latency, sleep duration, habitual sleep efficiency, sleep disturbance and daytime dysfunction, which were more obvious than those without LUTS/BPH. Among them, the difference of habitual sleep efficiency between the two groups was the most obvious. The above results have significant statistical significance (*P* < 0.005).

In addition, it was found that sleep disorders were associated with an increased prevalence of LUTS/BPH. As a sensitivity analysis, we excluded daytime LUTS/BPH analysis after nocturia, similarly, sleep disorders were positively correlated with increased prevalence of daytime LUTS/BPH (Table 2), further enhancing the robustness of our study.

**TABLE 2:** Associations between sleep disorder and LUTS/BPH and daytime LUTS/BPH.

	Non-adjusted OR (95%CI)	*P*-value	Adjusted I OR (95%CI)	*P*-value	Adjusted II OR (95%CI)	*P*-value

***N***	**11,824**		**11,623**		**9,266**	
**LUTS/BPH**						
**Sleep disorder**						
No	1		1		1	
Yes	1.427 (1.298, 1.570)	<0.001	1.372 (1.245, 1.512)	<0.001	1.257 (1.119, 1.411)	<0.001
Global PSQI score	1.043 (1.029, 1.057)	<0.001	1.037 (1.023, 1.051)	<0.001	1.021 (1.004, 1.038)	0.013
**Daytime LUTS/BPH**						
**Sleep disorder**						
No	1		1		1	
Yes	1.605 (1.458, 1.767)	<0.001	1.534 (1.390, 1.691)	<0.001	1.343 (1.193, 1.512)	<0.001
Global PSQI score	1.072 (1.058, 1.086)	<0.001	1.064 (1.050, 1.078)	<0.001	1.044 (1.026, 1.061)	<0.001

Non-adjusted: no covariates were adjusted.

Adjusted I: adjusted for age.

Adjusted II: adjusted for age, BMI, educational level, marital status, smoking status, drinking status, coffee intake, tea intake, WHR, PHQ-9, GAD-7, comorbidity index, DM, physical activity, Cr.

The results of logistic regression analyses of the correlation between global PSQI and its seven components and LUTS/BPH prevalence among participants were shown in Table 3. In the crude model, the global PSQI score (≤7 or >7) was positively correlated with the prevalence of LUTS/BPH. After adjusting for confounding variables, the global PSQI score (≤7 or >7) was still significantly positively correlated with the prevalence of LUTS/BPH (OR: 1.021; 95% CI: 1.004–1.038; *P* = 0.013). Similar results can be observed in the seven components of PSQI. We found that among the six components other than use of sleep drugs, the higher PSQI components score, the more significant positive correlation between sleep quality and the risk of LUTS/BPH (*P* < 0.05).

**TABLE 3:** Regression analysis between PSQI components and LUTS/BPH regression analysis.

	Non-adjusted OR (95%CI)	*P*-value	Adjusted I OR (95%CI)	*P*-value	Adjusted II OR (95%CI)	*P*-value	*P* for trend
**PSQI domain scores**							
Subjective sleep quality							<0.001
0	1		1		1		
1	1.197 (1.072, 1.336)	0.001	1.163 (1.040, 1.300)	0.008	1.130 (0.995, 1.284)	0.060	
2	1.657 (1.440, 1.906)	<0.001	1.635 (1.418, 1.886)	<0.001	1.535 (1.299, 1.814)	<0.001	
3	1.931 (1.494, 2.497)	<0.001	1.800 (1.384, 2.342)	<0.001	1.376 (1.004, 1.886)	0.048	
Sleep latency							<0.001
0	1		1		1		
1	0.991 (0.887, 1.107)	0.872	1.000 (0.894, 1.118)	0.994	0.893 (0.783, 1.018)	0.089	
2	0.565 (0.507, 0.630)	<0.001	0.603 (0.540, 0.673)	<0.001	0.500 (0.439, 0.569)	<0.001	
3	0.808 (0.705, 0.926)	0.002	0.833 (0.726, 0.957)	0.010	0.656 (0.557, 0.773)	<0.001	
Sleep duration							<0.001
0	1		1		1		
1	1.143 (1.037, 1.260)	0.007	1.137 (1.030, 1.255)	0.011	1.120 (0.999, 1.255)	0.052	
2	1.396 (1.214, 1.604)	<0.001	1.276 (1.107, 1.470)	0.001	1.218 (1.035, 1.432)	0.017	
3	1.710 (1.454, 2.012)	<0.001	1.537 (1.303, 1.814)	<0.001	1.441 (1.189, 1.745)	<0.001	
Habitual sleep efficiency							<0.001
0	1		1		1		
1	1.171 (1.049, 1.308)	0.005	1.099 (0.982, 1.230)	0.099	1.135 (0.999, 1.289)	0.051	
2	1.487 (1.307, 1.692)	<0.001	1.362 (1.194, 1.554)	<0.001	1.306 (1.123, 1.520)	0.001	
3	1.650 (1.471, 1.849)	<0.001	1.451 (1.289, 1.632)	<0.001	1.369 (1.193, 1.570)	<0.001	
Sleep disturbance							0.002
0	1		1		1		
1	1.217 (1.069, 1.384)	0.003	1.104 (0.967, 1.259)	0.143	1.022 (0.881, 1.184)	0.777	
2	1.898 (1.595, 2.259)	<0.001	1.686 (1.411, 2.013)	<0.001	1.354 (1.102, 1.664)	0.004	
3	3.022 (1.520, 6.011)	0.002	2.568 (1.283, 5.141)	0.008	2.539 (1.169, 5.518)	0.019	
Use of sleep drugs							0.086
0	1		1		1		
1	1.252 (0.770, 2.035)	0.365	1.216 (0.745, 1.985)	0.435	1.152 (0.648, 2.050)	0.629	
2	1.477 (0.866, 2.520)	0.152	1.347 (0.784, 2.313)	0.281	1.149 (0.596, 2.218)	0.678	
3	1.706 (1.113, 2.615)	0.014	1.497 (0.972, 2.306)	0.067	1.517 (0.927, 2.481)	0.097	
Daytime dysfunction							<0.001
0	1		1		1		
1	0.970 (0.858, 1.097)	0.631	1.111 (0.979, 1.260)	0.103	1.084 (0.935, 1.256)	0.286	
2	1.033 (0.871, 1.226)	0.710	1.196 (1.003, 1.425)	0.046	1.152 (0.942, 1.408)	0.168	
3	1.603 (1.253, 2.050)	<0.001	1.890 (1.469, 2.431)	<0.001	1.702 (1.278, 2.267)	<0.001	

Non-adjusted: no covariates were adjusted.

Adjusted I: adjusted for age.

Adjusted II: adjusted for age, BMI, educational level, marital status, smoking status, drinking status, coffee intake, tea intake, WHR, PHQ-9, GAD-7, comorbidity index, DM, physical activity, Cr.

**FIGURE 3:**
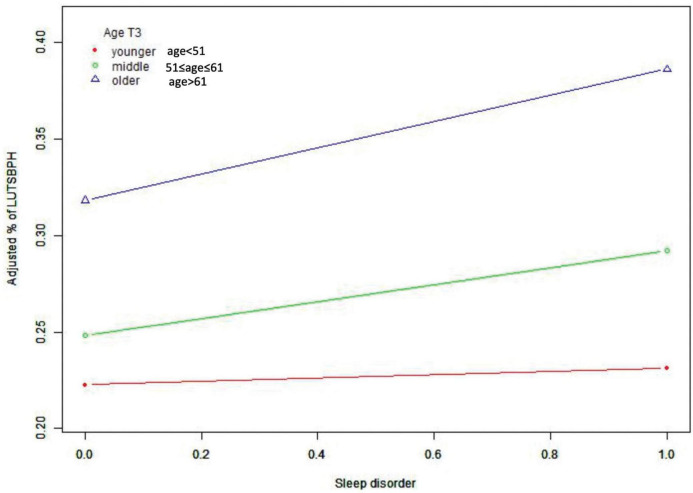
Smooth curve fitting of sleep disorder and risk of LUTS/BPH, grouped by age.

**FIGURE 4:**
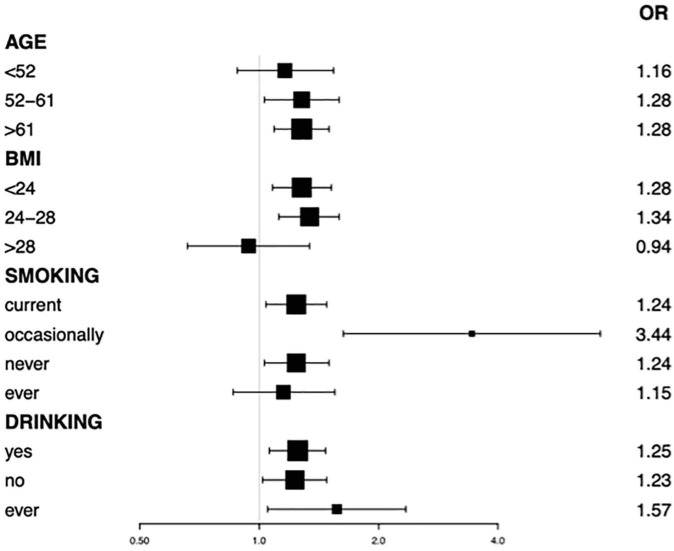
Subgroup analysis by age, BMI, smoking, drinking status on association between PSQI with LUTS/BPH.

Subgroup analysis stratified by the age of participants also found a positive correlation between sleep quality and the prevalence of LUTS/BPH (Table 4). We observed no statistically significant association of sleep quality and LUTS/BPH among young-aged male participants. On the other hand, among the middle and older-aged male participants, higher PSQI score (>7) were significantly associated with the increased prevalence of LUTS/BPH in all models (51 ≤ middle-aged ≤ 61: OR: 1.277; 95%Cl: 1.026–1.589; *P* = 0.029) (older-aged > 61: OR: 1.277; 95%Cl: 1.088–1.499; *P* = 0.003) (Figure 3). Meanwhile, we observed a significant interaction between sleep quality and LUTS/BPH in the correlation of age in the three models (*p* for interaction = 0.005). In addition, we further carried out subgroup analysis (Figure 4). The results of subgroup analysis showed that our findings were significant in most subgroups (*P* < 0.05), reflecting the stability of the results. Only in the age < 52 group and BMI > 28 group, we did not observe significant results.

**TABLE 4:** Stratified multiple logistic regression analysis to identify variables that modify the correlation between sleep quality and LUTS/BPH.

Subgroup		Non-adjusted OR (95%CI)	*P*-value	Adjusted I OR (95%CI)	*P*-value	Adjusted II OR (95%CI)	*P*-value
Age-young	Global PSQI score	1.007 (0.976, 1.039)	0.651	0.999 (0.967, 1.032)	0.946	0.996 (0.955, 1.039)	0.863
	**Sleep disorder**						
	No	1		1		1	
	Yes	1.198 (0.956, 1.500)	0.116	1.166 (0.927, 1.468)	0.189	1.161 (0.876, 1.539)	0.300
Age-middle	Global PSQI score	1.014 (0.988, 1.040)	0.290	1.010 (0.984, 1.036)	0.457	1.005 (0.974, 1.037)	0.778
	**Sleep disorder**						
	No	1		1		1	
	Yes	1.361 (1.133, 1.635)	<0.001	1.334 (1.108, 1.605)	0.002	1.277 (1.026, 1.589)	0.029
Age-older	Global PSQI score	1.061 (1.042, 1.080)	<0.001	1.061 (1.042, 1.080)	<0.001	1.036 (1.013, 1.059)	0.002
	**Sleep disorder**						
	No	1		1		1	
	Yes	1.465 (1.282, 1.675)	<0.001	1.456 (1.272, 1.668)	<0.001	1.277 (1.088, 1.499)	0.003
Total	PSQI	1.038 (1.024, 1.052)	<0.001	1.035 (1.022, 1.049)	<0.001	1.022 (1.005, 1.039)	0.009
	**PSQI domain scores**						
	≤7	1		1		1	
	>7	1.381 (1.253, 1.522)	<0.001	1.362 (1.234, 1.503)	<0.001	1.264 (1.126, 1.419)	<0.001
*P* interaction		0.002		<0.001		0.005	

Age-young: aged ≤ 51.

Age-middle: 51 < aged < 61.

Age-older: aged ≥ 61.

Non-adjusted: no covariates were adjusted.

Adjusted I: adjusted for age.

Adjusted II: adjusted for age, BMI, educational level, marital status, smoking status, drinking status, coffee intake, tea intake, WHR, PHQ-9, GAD-7, comorbidity index, DM, physical activity, Cr.

## Discussion

With the continuous development of population aging trend, it is estimated that by 2050, the population over 65 years old will reach 400 million (accounting for 26.9% of the total population), and the population over 80 years old can grow to 150 million (Fang et al., 2015). More evidence shows that LUTS/BPH is the manifestation of urinary system caused by multiple systems (Fantus et al., 2018). The purpose of this study was to further clarify the correlation between sleep disorders and LUTS/BPH in Chinese males. The data come from a large natural population cohort represented by people in Southwest China. This study showed that poor sleep quality was significantly associated with the prevalence of LUTS/BPH in men. In the subgroup analysis, the fully adjusted model results showed that the significant association between poor sleep quality and the prevalence of LUTS/BPH mainly existed in male participants aged ≥ 51. There was a significant interaction between age and sleep quality and the prevalence of LUTS/BPH.

The results of a prospective cohort study conducted by Araujo et al. (2014) showed that the correlation between sleep disorders and urinary symptoms is bi-directional. Similar results were also observed in the study of Scovell et al. (2017), which also found that men with sleep difficulties, difficulty in maintaining sleep and difficulty in returning to sleep reported more serious LUTS than men without similar sleep difficulties. The results of the above articles show that sleep problems are associated to male LUTS/BPH, which is consistent with the results of this study.

There are several hypotheses about the biological basis of sleep and its correlation with LUTS/BPH. One theory involves the role of circadian rhythm in hormone metabolism. Sleep structure is usually affected by the aging process: it becomes more and more fragmented due to the arousals and episodes of awakening, which may lead to poor sleep maintenance and consolidation, as well as the reduction of the overall amount of sleep (Kryger et al., 2004). Now emerging evidence shows that similar partial sleep deprivation patterns can affect many aspects of mammalian endocrine system, including the decline of some anabolic hormones (Everson and Crowley, 2004). Age related sleep fragmentation may disrupt androgen secretion in older men (Luboshitzky et al., 2001). Copinschi and Caufriez (2013) found that circadian rhythm of circulating testosterone seems to be mainly driven by the sleep wake cycle. In fact, daytime and nighttime sleep are associated with strong increases in circulating testosterone levels. However, although it is weakened compared with night sleep, testosterone levels continue to rise when awake at night. Nocturnal sleep time has been shown to be an independent predictor of morning total and free testosterone in the elderly. Therefore, with the duration and length of sleep time, it has a great impact on the fluctuation of hormone level in the body, especially testosterone, so LUTS/BPH dependent on androgen level is more likely to occur. Related studies put forward a possibility that the change of sleep may be one of the mechanisms that transform the physiological aging process of the elderly into the change of neuroendocrine function (Penev, 2007). In addition, reduced sleep duration can cause the disorders of circadian clock genes, such as *Per 2*, *Per 3* (Goel et al., 2013). Meanwhile, in the research results reported by Li et al. (2019), the decreased expression of *Per 2* can inhibit cell apoptosis, leading to BPH. Therefore, good sleep is of great significance to maintain the stability of the circadian rhythm.

Another theory suggested that autonomic nerve activity may be one of the causes of LUTS/BPH in men. Roehrborn (2008) found that autonomic nervous system activity, plasma and urinary catecholamines were positively correlated with symptom scores and other BPH measurements. Meanwhile, previous studies by Spiegel et al. (1999) showed that the activity of sympathetic nervous system increased under the condition of sleep debt (*p* < 0.02). Thus, sleep disorder may affect the pathological changes of prostate by promoting the activity of sympathetic nerve, so as to cause the occurrence of LUTS. In addition, the latest research results suggested that prostatic hyperplasia and pathological progression are related to the level of inflammation, and sleep disorders can increase the level of inflammatory factors in the body (Navaneethakannan et al., 2022).

Subgroup analysis showed that the association between increased sleep disorder incidence and increased prevalence of LUTS/BPH was much more common among older male participants. Poor sleep has many negative effects on quality of life and increases the risk of other comorbidity such as depression, obesity, type 2 diabetes, cardiovascular disease, which may have a negative impact on mood, subjective well-being and overall function (Araujo et al., 2014; Scovell et al., 2017). For the elderly, the risk of comorbidity caused by sleep disorders will increase, which is more likely to lead to the occurrence of LUTS/BPH.

It should be noted that there are several limitations of this study. First, due to the cross-sectional nature of this study, the causal association between sleep quality and LUTS/BPH may not be clarified, and further prospective and intervention studies may provide a better explanation. Secondly, although we adjusted for potential confounding factors, we still can not rule out the impact of other unmeasured correlation factors on the results. What is more, because the diagnostic information of LUTS/BPH is self-reported, there is a risk of bias. Finally, there may be recall bias for some variables (such as smoking, drinking, exercise variables, etc.).

## Conclusion

Our findings suggested that sleep disorders are associated with an increased prevalence of LUTS/BPH. Moreover, the significant interaction between age and sleep disorders showed that older people are more likely to develop LUTS/BPH due to sleep disorders. Our study provides data support for the possible future proposal to prevent LUTS/BPH by improving sleep disorders. Their potential biological mechanisms need to be further studied.

## Data availability statement

The raw data supporting the conclusions of this article will be made available by the authors, without undue reservation.

## Ethics statement

The studies involving human participants were reviewed and approved by Biomedical Ethics Review Committee of West China Hospital of Sichuan University. The patients/participants provided their written informed consent to participate in this study. Written informed consent was obtained from the individual(s) for the publication of any potentially identifiable images or data included in this article.

## Author contributions

YL contributed to the design of the study, was responsible for data processing and analysis, and drafted the initial manuscript. SQ, XZ, and KJ decided the methods of data analysis. LC, ZJ, DH, and MW were responsible for determining whether the data should be included in the discharge criteria. SW and BC polished the manuscript. QW, LY, and LM conceptualized and designed the study, supervised all aspects of the study, critically reviewed and revised the manuscript, and approved the final manuscript submitted. All authors read and approved the final manuscript.

## Footnotes

1
http://www.R-project.org

2
http://www.empowerstats.com

## Abbreviations

BPH: benign prostatic hyperplasia
BOO: bladder outlet obstruction
BMI: body mass index
Cr: creatinine
DM: diabetes mellitus
GAD-7: generalized anxiety disorder-7
LUTS: lower urinary tract symptoms
MECs: mobile examination centers
PSQI: Pittsburgh Sleep Quality Index
PHQ-9: patient health questionnaire-9
WCNPCS: West China Natural Population Cohort Study
WHR: waist-to-hip ratio.
